# Preventive Behaviors Among Leukemia and Lymphoma Cancer Survivors: Results From the 2020 Behavioral Risk Factor Surveillance System Survey

**DOI:** 10.1016/j.focus.2022.100041

**Published:** 2022-10-24

**Authors:** Steven S. Coughlin, Biplab Datta, Ban Majeed

**Affiliations:** 1 Department of Population Health Sciences, Augusta University, Augusta, Georgia;; 2 Institute of Public and Preventive Health, Augusta University, Augusta, Georgia

## Abstract

**Introduction:**

Maintaining a healthy lifestyle is an important factor in promoting positive outcomes for cancer survivors. Health behaviors, such as engaging in physical activity, not consuming large amounts of alcohol, abstaining from cigarette smoking, receiving routine immunizations, and undergoing recommended cancer screening tests, may lead to improved physical health, health status, and quality of life.

**Methods:**

We examined preventive behaviors among hematologic cancer survivors in a cross-sectional analysis, using data from the 2020 Behavioral Risk Factor Surveillance System.

**Results:**

Adherence to cancer screening test and immunization recommendations among hematologic cancer survivors compares favorably with that among persons with no history of cancer. However, no statistically significant differences in the frequency of current smoking, heavy drinking, and physical activity were observed across these 2 groups. No important differences were observed in health behaviors between male and female cancer survivors, except that female hematologic cancer survivors were more likely to adhere to influenza and pneumococcal pneumonia immunization recommendations than female survivors of other types of cancer, whereas no such differences were found among their male counterparts.

**Conclusions:**

Although hematologic cancer survivors were more adherent to preventive health behaviors such as cancer screening and immunization, they were not different from persons without any history of cancer in exhibiting behavioral risk factors such as smoking and heavy drinking. Intervention studies are needed to identify effective ways to assist hematologic cancer survivors to quit smoking and refrain from hazardous alcohol consumption.

## INTRODUCTION

Lymphoma is a heterogeneous disease categorized into 2 main types: Hodgkin lymphoma and non-Hodgkin lymphoma. Non-Hodgkin lymphoma is the seventh most common cancer in the U.S., with 19.6 new cases per 100,000 per year in 2014–2018.^1^ As of January 1, 2019, it is estimated that there were 757,710 non-Hodgkin lymphoma survivors in the U.S.^2^ With improvement in therapies and supportive care, the numbers of lymphoma survivors continue to grow. Leukemia, which is also a heterogeneous disease, is the ninth most common cancer in the U.S., with 14.3 new cases per 100,000 persons per year in 2014–2018.^1^ As of January 1, 2019, it is estimated that there were 451,700 leukemia survivors in the U.S.^2^

Maintaining a healthy lifestyle is an important factor in promoting positive outcomes for cancer survivors, including hematologic cancer survivors. Health behaviors, such as engaging in physical activity, not consuming large amounts of alcohol, abstaining from cigarette smoking, receiving routine immunizations, and undergoing recommended cancer screening tests, may improve health and quality of life.^3,4^ For example, physical activity and avoiding smoking reduce the risk of cancer recurrence, secondary cancers, and chronic illness (e.g., cardiovascular disease, diabetes).^5^ However, most of the published literature on preventive behaviors among cancer survivors has focused on cancer of the breast and on certain other cancers that are mostly tobacco related. Hematologic cancer survivors often have prolonged immunosuppression status and a higher risk of infections.^6^ Thus, immunization status is an important consideration when considering health behaviors.

Cancer survivors who have completed primary therapy for the disease often have complex healthcare needs that include close follow-up to detect recurrence and second primary malignancies and the management of cancer-related morbidities. Appropriate care during the post-initial treatment survivorship phase includes preventive care visits and screening for cancer recurrence.

We examined preventive behaviors among hematologic cancer survivors (non-Hodgkin lymphoma, Hodgkin lymphoma, leukemia) using data from the 2020 Behavioral Risk Factor Surveillance System (BRFSS). We compared behavioral risk factors and preventive measures among male and female hematologic cancer survivors, survivors of other types of cancer, and men and women without a history of cancer. Our hypothesis was that the prevalence of preventive measures among hematologic cancer survivors would be similar to that among survivors of other types of cancer and higher than the prevalence among men and women without a history of cancer.

## METHODS

### Study Sample

BRFSS is a cross-sectional telephone-based survey of U.S. residents aged ≥18 years, which collects information about health-related risk factors, chronic health conditions, and the use of preventive services.^7^ Cancer survivor data were available for 21 states (Arizona, Connecticut, Delaware, Georgia, Hawaii, Indiana, Louisiana, Massachusetts, Michigan, Mississippi, Missouri, Montana, New Jersey, New Mexico, North Carolina, Rhode Island, South Dakota Utah, Vermont, Virginia, Wisconsin). The mean and median combined (landline and cell phone) weighted response rates were 47.12% and 46.80%, respectively.

### Measures

The following health behaviors were assessed in this study: receipt of recommended cancer screening tests, including colorectal cancer screening among adults aged >45 years and breast cancer screening (mammogram) among women aged >40 years; vaccinations for influenza (among adults aged >18 years) and pneumonia (among adults aged >65 years); current smoking status and alcohol consumption; and participation in physical activities or exercises. Colorectal cancer screening was assessed according to the U.S. Preventive Services Task Force recommendations as the receipt of any of the following: colonoscopy within 10 years, virtual colonoscopy within 5 years, sigmoidoscopy within 5 years, stool DNA test within 3 years, and blood stool test within 1 year.^8^ Breast cancer screening was assessed according to U.S. Preventive Services Task Force recommendations as the receipt of a mammogram in the past 2 years. We determined the status of up-to-date influenza and pneumococcal vaccinations with the following survey questions: *During the past 12 months, have you had either flu vaccine sprayed in nose or flu shot injected into arm?* and *Have you ever had a pneumonia shot/pneumococcal vaccine?* Current smokers were defined as respondents who smoked at least 100 cigarettes in their lifetime and now smoke some days or every day. Heavy alcohol use was defined as an average intake of 14 drinks per week for a man and 7 drinks per week for a woman. Finally, a respondent was determined as physically active if they answered affirmative to the following question: *During the past month*… *did you participate in any physical activities such as running, calisthenics, golf, gardening or walking for exercise?*

In the BRFSS, respondents were asked whether they were ever told that they have any type of cancer (other than skin cancer). Respondents who answered *no* to this question were regarded as without any history of cancer. The cancer survivorship module of the 2020 BRFSS further asked respondents with a history of cancer about their type of cancer. The survey reported 28 different types (excluding nonmelanoma skin cancer) of cancer, from which we were able to identify hematologic and other cancer survivors. Consequently, we divided the sample into 3 mutually exclusive groups: without cancer, survivors of hematologic cancer, and survivors of other types of cancer. We examined how the percentage of respondents displaying certain preventive health behaviors and behavioral risk factors varies across these groups. Adjusted Wald tests were performed to examine whether the shares differ between survivors of hematologic and other cancers.

### Statistical Analysis

We then fit a logistic regression model for each behavior/risk factor to estimate the odds in favor of displaying the respective behavior/risk factor for each mutually exclusive group. The without cancer category served as the reference group because we examined how the odds differ for the hematologic cancer survivors and the other cancer survivors compared with the odds for those having no history of cancer. To obtain adjusted ORs, we used multiple logistic regression to control for age, race/ethnicity, educational attainment, household income, marital status, and employment status. Further, to account for geographic differences, we controlled for urban/rural location and state of residence fixed effects.

Age, in the multivariable model, was controlled as a polychotomous variable with the following categories: ages 18–49 (ref), 50–64, and ≥65 years. Race, education, income, marital status, and employment status were also controlled this way. Race and ethnicity comprised 4 categories: non-Hispanic White (ref), non-Hispanic Black, Hispanic, and non-Hispanic other. Education groups were less than high school diploma (ref), high school graduate, some college, and college graduate. Income categories were <$15,000 (ref), $15,000–$24,999, $25,000–$34,999, $35,000–$49,999, and ≥$50,000. Marital status was controlled for not married (ref), married, and maritally disrupted (i.e., widowed, divorced, or separated). Finally, employment status categories were employed (ref), not employed (including out of work, homemaker, student, and unable to work), and retired. All analyses were conducted with Stata, Version 17.0, and used BRFSS sampling weights to adjust for the complex sampling design.

## RESULTS

A total of 695 hematologic cancer survivors were identified, along with 21,868 survivors of other types of cancer and 135,916 respondents without cancer. Characteristics of the study sample by population subgroup are shown in Table 1. About 59.2% were male. About half (50.0%) were aged ≥65 years. About 81.6% were White, and the remainder were Black (8.9%), Hispanic (1.6%), or of other race (8.0%). About 10.4% had less than a high school education. Almost half (47.2%) had an income of at least $50,000 per year.

Table 2 shows the frequency of preventive health behaviors and behavioral risk factors among hematologic cancer survivors, survivors of other types of cancer, and respondents with no history of cancer. Among men and women aged ≥45 years, a similar proportion of hematologic cancer survivors and survivors of other types of cancer adhered to recommendations for routine colorectal cancer screening (Table 2). The proportion of hematologic cancer survivors who adhered to recommendations for colorectal cancer screening was higher than that of respondents without a history of cancer. Among women aged ≥40 years, the proportion of hematologic cancer survivors who adhered to recommendations for breast cancer screening was higher than that of respondents without a history of cancer (83.18% vs 71.05%, respectively, *p*<0.001). Among men, a similar proportion of hematologic cancer survivors and survivors of other types of cancer adhered to recommendations for influenza immunization. The proportion of male hematologic cancer survivors who adhered to recommendations for influenza immunization was higher than that of respondents without a history of cancer (65.12% vs 40.82%, respectively, *p*<0.001). Among women, the proportion of hematologic cancer survivors who adhered to recommendations for influenza immunization was higher than that of respondents with a history of other types of cancer (*p*=0.021) and those without a history of cancer (*p*<0.001).

The adherence to pneumococcal pneumonia immunization was markedly higher (*p*<0.001) among male (age ≥65 years) hematologic cancer survivors and survivors of other types of cancer than among those without any history of cancer; however, the difference between the 2 cancer survivor groups was not statistically significant. Adherence in women, by contrast, was significantly higher (*p*<0.001) among hematologic cancer survivors than among both other cancer survivors and respondents having no cancer. Although the frequency of current smoking was lower among other cancer survivors of both sexes than the frequency among those with no cancer, the rates among survivors of hematologic cancer were not statistically different from those of respondents without any history of cancer. The pattern for heavy drinking among male respondents was similar. Among females, no significant differences were observed in heavy drinking across the groups. Finally, there was no significant difference in the frequency of physical activity between the hematologic cancer survivors and respondents without cancer; although the frequency was lower (*p*<0.001) for other cancer survivors of both sexes than for those who reported no history of cancer (Table 2). When comparisons were made between hematologic and other cancer survivors, no significant differences were observed except for the influenza vaccination among females. Frequency of influenza vaccination was 9.2 percentage points higher (*p*=0.021) among female hematologic cancer survivors than among female survivors of other types of cancer.

The unadjusted ORs and AORs in favor of displaying certain preventive behaviors or behavioral risk factors for the mutually exclusive groups are presented in Table 3. The odds of cancer screening and vaccination were higher (*p*<0.001) among survivors of hematologic cancer and other types of cancer than among those without any history of cancer. The results persisted after adjusting for various socio-demographic confounders. However, none of the adjusted odds were statistically significant for tobacco or alcohol consumption indicators. The odds of being physically active were lower for those with other types of cancer and were not statistically significant for both male and female survivors of hematologic cancer (Table 3). Results of the adjusted Wald tests suggested that none of the odds for hematologic and other cancer indicators were statistically different from each other except for influenza and pneumonia vaccination among females.

Results (AORs) by urban and rural subgroups are presented in Table 4. The higher likelihood of colorectal cancer screening among hematologic and other cancer survivors was evident in the urban subsample. In the rural subsample, the adjusted odds of colorectal cancer screening, however, were not statistically significant for hematologic cancer survivors. Unlike the estimates in the full sample and urban subsample, the adjusted odds of influenza vaccination for the male hematologic cancer survivors were not statistically significant in the rural subsample. Similarly, the higher odds of pneumonia vaccination for the female hematologic cancer survivors did not persist in the rural subsample. Finally, in contrast to the findings in the full sample and urban subsample, male hematologic cancer survivors in the rural subsample showed significantly higher odds of smoking. By contrast, female hematologic cancer survivors in the rural subsample had a lower likelihood of smoking than their counterparts with no history of cancer as well as survivors of other types of cancer.

## DISCUSSION

The results of this population-based study indicate that adherence to cancer screening and immunization recommendations among hematologic cancer survivors compares favorably with that of persons with no history of cancer. The adherence to cancer screening was not different between hematologic survivors and survivors of other types of cancer. Adherence to immunization, by contrast, was higher among female hematologic cancer survivors than among female survivors of other types of cancer. However, no such differences across these 2 groups were found among male cancer survivors. Other than the difference in adherence to influenza and pneumococcal pneumonia immunization recommendations mentioned earlier, no differences were observed in cancer screening and behavioral risk factors between male and female cancer survivors. Of note, despite the fact that significant differences were observed between hematologic cancer survivors and individuals with no history of cancer, no significant differences, in general, were observed between survivors of hematologic and other types of cancer. However, some interesting heterogeneities were observed in the urban and rural subgroups.

In this study, 10.8%–14.5% of hematologic cancer survivors are current cigarette smokers, and 5.1%–7.2% are heavy alcohol drinkers, thus at higher risk of cancer recurrence, secondary malignancies, and other chronic diseases. Oncologists and primary care providers have important roles in encouraging cancer survivors to reduce their risk of serious illness or death through healthy lifestyle changes.

This study showed that the prevalence of current cigarette smokers (having smoked ≥100 cigarettes in a lifetime and smoked every day or some days at the time of the survey) was 17.5% among adults who had no history of any cancer diagnosis, 14.5% in adults with hematologic cancer, and 12.5% in adults with other types of cancer. These rates are comparable with national cigarette smoking rates. Data from the 2018 National Health Interview Survey, an annual, nationally representative household survey of the non-institutionalized U.S. civilian population, revealed that of U.S. adults, 13.7% (13.1–14.3) were current cigarette smokers.^9^ Differences in national estimates may be attributed to slight methodologic and timing differences in these national surveys.

In this study, results of bivariate analyses showed that current smoking rates were significantly higher among adults with no history of cancer than among the other 2 groups with hematologic and other types of cancer. These differences were not statically significant after controlling for other personal characteristics, that is, the adjusted regression models. We offer 3 possible explanations for the observed lower rates of cigarette smoking in the 2 groups with a history of cancer than in adults with no history of cancer. First, given the stigma associated with reporting cigarette smoking status,^10,11^ especially among patients with cancer,^12^ these rates could be an underestimate resulting from under-reporting of smoking status. Second, lower rates of current smoking in adults with a history of cancer could be the result of successful quitting after cancer diagnoses and treatment.^13^ Continued smoking after cancer diagnosis is associated with increased treatment-related toxicity, complications, and poor long-term health outcomes; thus, smoking cessation is central to comprehensive cancer treatment and care, which in turn could continue to lower the rates of current cigarette smoking in patients with a history of cancer.^14^ Third, the question used in this national survey reflects 1 type of tobacco use, cigarettes, thus not considering the use of noncigarette tobacco products, for example, smokeless tobacco, associated with oral and esophageal cancers.^15^ However, the odds of smoking among male hematologic cancer survivors in the rural areas were found to be significantly higher than those of their counterparts without any history of cancer. Nevertheless, the odds were not statistically different from those of male cancer survivors of other types in the rural areas. These results show the need for further nuanced analysis by cancer type and geographic location to augment cancer prevention efforts.

### Limitations

With respect to limitations, we were unable to examine healthy eating because the 2020 BRFSS questionnaire did not include a food frequency questionnaire or ask about servings of fruits and vegetables consumed. Because BRFSS is a telephone survey, the estimates obtained in this study may not apply to persons who lack a household telephone. The results may not be generalizable to all hematologic cancer survivors in the U.S. because of the low response rate in the BRFSS and because not all states were represented. In addition, misclassification bias may have occurred owing to the use of self-reported information. The definition of physically active was broad in nature, which kept us from doing a more nuanced analysis of this lifestyle factor. We are limited by a lack of data on temporality, including pre-diagnosis behaviors. Furthermore, the BRFSS does not collect any information about cancer characteristics (e.g., stage at diagnosis), phase of care, treatment patterns, or cancer incidence. The lack of data about medication adherence is a further study limitation.

Apart from some heterogeneities across rural and urban subsamples, in general, we did not observe any significant differences in preventive behaviors between survivors of hematologic and other types of cancer. Individuals with no history of cancer showed relatively lower odds of preventive screening and higher odds of some risky health behaviors than these 2 groups. As such, in addition to promoting preventive health services for cancer survivors, there is a need to boost cancer prevention efforts in the general population.

## CONCLUSIONS

Adherence to cancer screening and immunization recommendations among hematologic cancer survivors compared favorably with that among those with no history of cancer. However, smoking and heavy drinking behaviors among hematologic cancer survivors were not different from those among persons without any history of cancer. Intervention studies are needed to identify effective ways to assist hematologic cancer survivors to quit smoking and refrain from hazardous alcohol consumption.^14,16^ In addition, recommendations for preventive behaviors should be mentioned in survivorship care plans.

## Figures and Tables

**Table 1.** Demographic and Socioeconomic Characteristics of Study Participants

Characteristics		Population subgroups		
	General population, *n* (%)	Respondents without cancer, *n* (%)	Respondents with other types of cancer, *n* (%)	Respondents with hematologic cancer, *n* (%)
Sex				
Male	71,996 (48.55)	62,425 (49.1)	9,217 (43.06)	354 (59.16)
Female	86,483 (51.45)	73,491 (50.9)	12,651 (56.94)	341 (40.84)
Age, years				
18–49	58,769 (52.5)	57,022 (56.9)	1,655 (12.33)	92 (21.99)
50–64	44,516 (25.42)	39,033 (25.04)	5,303 (28.94)	180 (28.00)
≥65	55,194 (22.08)	39,861 (18.06)	14,910 (58.74)	423 (50.01)
Race				
White	112,833 (64.87)	93,276 (62.67)	18,971 (84.89)	586 (81.56)
Black	14,254 (14.18)	13,302 (15)	921 (6.7)	31 (8.87)
Hispanic	13,031 (10.9)	12,450 (11.75)	558 (3.31)	23 (1.60)
Other	18,360 (10.04)	16,887 (10.57)	1,418 (5.11)	55 (7.96)
Education				
Less than high school	10,220 (11.41)	9,118 (11.58)	1,073 (9.9)	29 (10.36)
High school	41,886 (28.4)	36,559 (28.6)	5,157 (26.45)	170 (31.31)
Some college	43,384 (30.84)	37,052 (30.68)	6,141 (32.45)	191 (27.97)
College	62,262 (29.34)	52,510 (29.14)	9,449 (31.2)	303 (30.36)
Income				
<$15,000	9,994 (6.39)	8,807 (6.42)	1,155 (6.09)	32 (4.59)
$15,000–$24,999	18,953 (11.89)	16,341 (11.86)	2,512 (12.08)	100 (16.07)
$25,000–$34,999	12,153 (7.35)	10,220 (7.3)	1,880 (7.91)	53 (3.04)
$35,000–$49,999	16,994 (10.29)	14,382 (10.22)	2,527 (11.00)	85 (10.40)
≥$50,000	68,568 (42.88)	58,650 (42.79)	9,620 (43.65)	298 (47.23)
Marital status				
Not married	34,504 (29.76)	32,529 (32.05)	1,891 (8.97)	84 (13.76)
Married	81,714 (50.5)	69,094 (49.39)	12,244 (60.62)	376 (58.5)
Divorced/widowed/separated	40,741 (19.74)	32,883 (18.57)	7,627 (30.41)	231 (27.74)
Employment				
Employed	79,084 (56.24)	72,639 (58.92)	6,230 (32.11)	215 (42.21)
Not employed	30,442 (24.12)	27,271 (24.84)	3,058 (17.83)	113 (17.11)
Retired	46,078 (19.64)	33,231 (16.25)	12,482 (50.06)	365 (40.68)
Health coverage (age 18–65 years)				
Yes	12,012 (14.24)	11,611 (14.66)	388 (6.7)	13 (5.95)
No	90,639 (85.76)	83,827 (85.34)	6,553 (93.3)	259 (94.05)

*Note:* Percentages, presented in square brackets, were obtained using complex survey weights.

**Table 2.** Frequency of Preventive Health Behaviors and Behavioral Risk Factors Among Hematologic Cancer Survivors, Survivors of Other Types of Cancer, and Respondents Without Cancer

Behavior or risk factor		Population subgroups			Difference Hematologic versus other, %-points (95% CI)
	General population, % (95% CI) [*n*]	Respondents without cancer, % (95% CI) [*n*]	Respondents with other types of cancer, % (95% CI) [*n*]	Respondents with hematologic cancer, % (95% CI) [*n*]	
Cancer screening					
Colorectal cancer					
Male (age ≥45 years)	64.12(63.39, 64.85)[44,541]	60.68(59.85, 61.50)[35,337]	80.67(79.34, 82.00)[8,894]	79.38(75.32, 83.44)[310]	−1.29(−8.21, 5.62)
Female (age ≥45 years)	66.68(66.02, 67.33)[57,340]	64.00(63.26, 64.74)[45,125]	78.09(76.85, 79.32)[11,907]	81.55(77.52, 85.58)[308]	3.47(−4.09, 11.02)
Mammogram					
Male	—	—	—	—	
Female (age ≥40 years)	72.16(71.56, 72.75)[62,488]	71.05(70.39, 71.72)[50,127]	77.45(76.19, 78.71)(12,050)	83.18(77.90, 88.45)[311]	5.73(−0.67, 12.13)
Vaccination					
Influenza					
Male	43.25(42.65, 43.85)[67,516]	40.82(40.18, 41.45)[57,975]	66.26(64.67, 67.85)[9,195]	65.12(59.10, 71.13)[346]	−1.14(−9.67, 7.38)
Female	51.07(50.50, 51.65)[81,339]	49.08(48.46, 49.69)[68,397]	65.98(64.58, 67.39)[12,603]	75.15(69.02, 81.29)[339]	9.17(1.36, 16.98)
Pneumonia					
Male (age ≥65 years)	68.00(66.96, 69.03)(21,046)	64.09(62.81, 65.37)[14,428]	76.94(75.24, 78.63)[6,424]	83.23(78.67, 87.79)[194]	6.29(−2.61, 15.19)
Female (age ≥65 years)	74.30(73.46, 75.14)[29,702]	71.99(70.99, 73.00)[21,434]	80.40(78.96, 81.85)[8,047]	88.53(83.98, 93.08)[221]	8.13(0.01, 16.25)
Tobacco/alcohol consumption					
Current smoking					
Male	17.08(16.59, 17.56)[68,306]	17.55(17.03, 18.07)[58,809]	12.51(11.26, 13.77)[9,146]	14.51(9.06, 19.96)[351]	2.00(−4.09, 8.09)
Female	13.34(12.94, 13.73)(82,093)	13.46(13.03, 13.89)[69,186]	12.44(11.46, 13.43)[12,567]	10.77(5.90, 15.64)[340]	−1.68(−7.72, 4.37)
Heavy drinking					
Male	7.06(6.74, 7.38) [66,306]	7.19(6.85, 7.53) [56,951]	5.75(4.87, 6.64) (9,010)	7.15(5.19, 9.11) [345]	1.40(−4.47, 7.27)
Female	6.21(5.94, 6.48) [79,871]	6.24(5.95, 6.53) [67,681]	6.08(5.36, 6.81) [12,456]	5.07(1.21, 8.92) [339]	−1.01(−4.99, 2.97)
Physical activity					
Physically active					
Male	79.51(79.03, 80.00)[71,854]	79.96(79.44, 80.47)[62,293]	74.83(73.38, 76.28)[9,207]	78.86(75.29, 82.43)[354]	4.03(−2.69, 10.75)
Female	75.34(74.85, 75.82)[86,355]	75.97(75.45, 76.48)[73,383]	70.17(68.81, 71.52)[12,632]	71.26(64.76, 77.77)[340]	1.09(−7.04, 9.23)

*Note:* Estimates were obtained using complex survey weights. None of the differences between hematologic cancer survivors and other cancer survivors were statistically significant except for influenza vaccination among females.

**Table 3.** Unadjusted ORs and AORs in Favor of Preventive Behaviors and Behavioral Risk Factors for Hematologic Cancer Survivors and Other Cancer Survivors

Behavior or risk factor	Unadjusted OR			AOR		
	No cancer	Other cancer, estimate (95% CI)	Hematologic cancer, estimate (95% CI)	No cancer, estimate (95% CI)	Other cancer, estimate (95% CI)	Hematologic cancer, estimate (95% CI)
Cancer screening						
Colorectal cancer						
Male (age ≥45 years)	1.000 (ref)	2.705*** **(2.465, 2.968)**	2.495*** **(1.646, 3.781)**	1.000 (ref)	1.636*** **(1.463, 1.829)**	1.940** **(1.218, 3.088)**
Female (age ≥45 years)	1.000 (ref)	2.004*** **(1.849, 2.173)**	2.487*** **(1.514, 4.084)**	1.000 (ref)	1.633*** **(1.484, 1.798)**	1.800* **(1.011, 3.207)**
Mammogram						
Female (age ≥40 years)	1.000 (ref)	1.399*** **(1.291, 1.516)**	2.014*** **(1.285, 3.157)**	1.000 (ref)	1.321*** **(1.200, 1.455)**	2.403** **(1.393, 4.144)**
Vaccination						
Influenza						
Male	1.000 (ref)	2.848*** **(2.637, 3.075)**	2.707*** **(1.871, 3.916)**	1.000 (ref)	1.376*** **(1.254, 1.511)**	1.610* **(1.078, 2.404)**
Female	1.000 (ref)	2.013*** **(1.880, 2.156)**	3.138*** **(2.079, 4.738)**	1.000 (ref)	1.263*** **(1.163, 1.371)**	2.002** **(1.247, 3.212)**
Pneumonia						
Male (age ≥65 years)	1.000 (ref)	1.869*** **(1.672, 2.089)**	2.780*** **(1.483, 5.214)**	1.000 (ref)	1.698*** **(1.496, 1.927)**	3.349*** **(1.832, 6.123)**
Female (age ≥65 years)	1.000 (ref)	1.596*** **(1.435, 1.776)**	3.004*** **(1.366, 6.607)**	1.000 (ref)	1.356*** **(1.192, 1.542)**	3.371* **(1.125, 10.106)**
Tobacco/alcohol consumption						
Current smoking						
Male	1.000 (ref)	0.672*** **(0.595, 0.759)**	0.798 (0.493, 1.291)	1.000 (ref)	0.948 (0.820, 1.095)	1.035 (0.591, 1.813)
Female	1.000 (ref)	0.914* **(0.827, 1.009)**	0.776 (0.417, 1.445)	1.000 (ref)	1.035 (0.919, 1.166)	0.829 (0.423, 1.626)
Heavy drinking						
Male	1.000 (ref)	0.788*** **(0.662, 0.938)**	0.994 (0.414, 2.385)	1.000 (ref)	1.004 (0.817, 1.232)	1.221 (0.472, 3.158)
Female	1.000 (ref)	0.974 (0.845, 1.122)	0.803 (0.356, 1.811)	1.000 (ref)	1.057 (0.897, 1.245)	1.001 (0.444, 2.254)
Physical activity						
Physically active						
Male	1.000 (ref)	0.745*** **(0.685, 0.811)**	0.935 (0.630, 1.388)	1.000 (ref)	0.868** **(0.781, 0.964)**	0.850 (0.540, 1.338)
Female	1.000 (ref)	0.744*** **(0.692, 0.800)**	0.785 (0.530, 1.162)	1.000 (ref)	0.795*** **(0.726, 0.871)**	0.952 (0.554, 1.636)

*Note:* Boldface indicates statistical significance

(***p<0.001, **p<0.05, *p<0.1).

Estimates were obtained using complex survey weights. The 95% CIs are presented in parentheses. Regressions were separately estimated for each preventive behavior and behavioral risk factor, educational attainment, and household income. The adjusted odds were obtained by controlling for age, race and ethnicity, marital status, employment status, urban/rural location, and state of residence fixed effects. Results of adjusted Wald tests indicated that none of the odds for hematologic cancer and other cancer were statistically different from each other for respective outcomes except for influenza and pneumonia vaccination among females.

**Table 4.** AORs in Favor of Preventive Behaviors and Behavioral Risk Factors for Hematologic Cancer Survivors and Other Cancer Survivors by Urban and Rural

Behavior or risk factor	AORs for urban subsample (95% CI)			AORs for rural subsample (95% CI)		
	No cancer	Other cancer	Hematologic cancer	No cancer	Other cancer	Hematologic cancer
Cancer screening						
Colorectal cancer						
Male (age ≥45 years)	1.000 (ref)	1.651*** **(1.464, 1.861)**	2.228** **(1.364, 3.641)**	1.000(ref)	1.444* **(1.077, 1.936)**	0.321(0.085, 1.214)
Female (age ≥45 years)	1.000 (ref)	1.692*** **(1.530, 1.872)**	1.974* **(1.038, 3.755)**	1.000(ref)	1.165 (0.895, 1.517)	0.767 (0.305, 1.932)
Mammogram						
Female (age ≥40 years)	1.000 (ref)	1.311*** **(1.183, 1.453)**	2.338** **(1.313, 4.162)**	1.000 (ref)	1.455** **(1.133, 1.869)**	3.814* **(1.140, 12.762)**
Vaccination						
Influenza						
Male	1.000 (ref)	1.404*** **(1.271, 1.550)**	1.620* **(1.066, 2.461)**	1.000 (ref)	1.174 (0.909, 1.516)	1.787 (0.540, 5.920)
Female	1.000 (ref)	1.265*** **(1.160, 1.379)**	1.937** **(1.178, 3.184)**	1.000 (ref)	1.236 (0.985, 1.551)	2.945* **(1.165, 7.445)**
Pneumonia						
Male (age ≥65 years)	1.000 (ref)	1.728*** **(1.509, 1.979)**	3.297*** **(1.748, 6.218)**	1.000 (ref)	1.332 (0.963, 1.842)	5.460* **(1.170, 25.475)**
Female (age ≥65)	1.000 (ref)	1.340*** **(1.168, 1.537)**	3.386* **(1.032, 11.107)**	1.000 (ref)	1.513** **(1.121, 2.042)**	3.686 (0.833, 16.311)
Tobacco/alcohol consumption						
Current smoking						
Male	1.000 (ref)	0.891 (0.766, 1.036)	0.938 (0.514, 1.712)	1.000 (ref)	1.521 (0.966, 2.394)	4.029* **(1.254, 12.949)**
Female	1.000 (ref)	0.984 (0.865, 1.118)	0.935 (0.469, 1.862)	1.000 (ref)	1.563** **(1.135, 2.154)**	0.027*** **(0.004, 0.186)**
Heavy drinking						
Male	1.000 (ref)	0.977 (0.793, 1.204)	1.291 (0.499, 3.340)	1.000 (ref)	1.343 (0.598, 3.016)	-
Female	1.000 (ref)	1.074 (0.906, 1.274)	1.035 (0.446, 2.399)	1.000 (ref)	0.836 (0.492, 1.422)	0.501 (0.060, 4.170)
Physical activity						
Physically active						
Male	1.000 (ref)	0.880* **(0.788, 0.984)**	0.774*** **(0.702, 0.852)**	1.000 (ref)	0.782 (0.565, 1.084)	0.800 (0.246, 2.606)
Female	1.000 (ref)	0.855 (0.530, 1.378)	0.926 (0.517, 1.661)	1.000 (ref)	1.062 (0.826, 1.364)	1.595 (0.608, 4.182)

*Note:* Boldface indicates statistical significance

(***p<0.001, **p<0.05, *p<0.1).

Estimates were obtained using complex survey weights. The 95% CIs are presented in parentheses. Regressions were separately estimated for each preventive behavior and behavioral risk factor, educational attainment, and household income. The adjusted odds were obtained by controlling for age, race and ethnicity, marital status, employment status, and state of residence fixed effects. None of the male hematologic cancer survivors in the rural subsample were heavy drinkers.
